# Non-neoplastic Lesions of Gallbladder Among Cholecystectomy Specimens of a Tertiary Care Center: A Descriptive Cross-sectional Study

**DOI:** 10.31729/jnma.5861

**Published:** 2021-10-31

**Authors:** Dilasma Ghartimagar, Manish Kiran Shrestha, Adarsh Jhunjhunwala, Arnab Ghosh, Sushma Thapa, Sudeep Regmi, Om Prakash Talwar

**Affiliations:** 1 Department of Pathology, Manipal College of Medical Science, Pokhara, Nepal; 2 Department of Radiology, Charak Memorial Hospital, Pokhara, Nepal

**Keywords:** *cholecystitis*, *cholelithiasis*, *gallbladder*, *metaplasia*, *pyloric*

## Abstract

**Introduction:**

Gallbladder diseases are prevalent worldwide and present with a diverse histopathological spectrum. Mucosal irritation and chronic inflammation is considered as an important etiological factor for the mechanical or functional dysfunction of emptying of the gallbladder. This study aims to find the prevalence of non-neoplastic lesions of gallbladder among cholecystectomy specimens of a tertiary care center.

**Methods:**

A descriptive cross-sectional study was conducted in the Department of Pathology, of a tertiary care center from January 2005 to December 2020. Ethical approval was taken from the Institutional Review Committee. All the patients who had undergone cholecystectomy procedures which showed non-neoplastic lesions were enrolled in the study. Convenient sampling was done. Statistical Package for Social Sciences version 21 and Microsoft Excel were used for data analysis. Point estimate at 95% confidence interval was calculated along with frequency and proportion for binary data.

**Results:**

Out of 4914 cholecystectomy specimens, 4852 (98.73%) (95% Confidence Interval= 98.4299.04) were non-neoplastic lesions. There were 1252 (25.8%) males and 3600 (74.2%) females with a male to female ratio of 1:2.87. Age ranged from 2 to 89 years with a mean age of 45±14.48 years. Gallbladder lesions were observed maximum in age group 41-50 years with 1200 (24.7%) cases. Among the non-neoplastic lesions, cholecystitis without any specific finding was the most common finding with 3028 (62.4%) cases followed by cholelithiasis with 1478 (30.5%) cases.

**Conclusions:**

The prevalence of non-neoplastic lesions of gallbladder is similar to other studies done in similar setings. Female predominance was noted in non-neoplastic lesions.

## INTRODUCTION

The gallbladder is one of the most frequently resected organs which presents with a wide spectrum of lesions ranging from congenital anomalies, cholelithiasis, inflammatory and non-inflammatory lesions to noninvasive and invasive neoplasms.^1,2^

Cholelithiasis is a common disorder affecting 10-20% of adult population in developed countries and its incidence progresses with increasing age.^1,3^ It can lead to a variety of histopathological changes in gallbladder mucosa such as acute and chronic inflammation, cholesterolosis, metaplasias, hydrops and mucocele.^1,4^

This study aims to find the prevalence of non-neoplastic lesions of gallbladder among cholecystectomy specimens of a tertiary care center.

## METHODS

This is a descriptive cross-sectional study conducted in the Department of Pathology, Manipal College of Medical Sciences, Pokhara over a period of 16 years, from January 2005 to December 2020. Ethical approval was taken from the institutional review board (Ref: 388). The resected specimens comprises of laparoscopic cholecystectomy, open cholecystectomy and other surgeries were included in the study. Neoplastic lesions of gallbladder recorded during this period were excluded from the study. Convenient sampling was done. All the cholestectomy specimen over the period of 16 years were taken and the sample size were obtained by the following calculation.

n = Z^2^ × p × q / e^2^

  = (1.96)^2^ × 0.5 × 0.5 / (0.02)^2^

  = 2401

where,

n = Sample size
Z = 1.96 at 95% confidence interval
p = prevalence taken as 50%
q = 1-p
e = margin of error, 2%

However, a final sample size of 4914 was taken for the study. The data were analyzed using Microsoft Excel and Statistical Package for Social Sciences version 21. Point estimate at 95% confidence interval and descriptive statistics were calculated.

## RESULTS

Out of 4914 cholecystectomy specimens, 4852 (98.73%) (95% confidence interval= 98.4299.04) were non-neoplastic lesions. Amongst the non-neoplastic lesions, there were 1252 males and 3600 females with a male to female ratio of 1:2.87 Age ranged from 2 to 89 years with a mean age of 45 ±14.48 years. Gallbladder lesions were observed maximum in age group 41-50 years with 1200 cases which was followed by age group 31-40 years with 979 cases and age group 51-60 years with 876 cases (Table 1).

**Table 1:** Gallbladder lesion distribution according to age group (n = 4852).

Age group	n (%)
0-10	130 (0.3)
11-20	153 (3.2)
21-30	818 (16.9)
31-40	979 (20.2)
41-50	1200 (24.7)
51-60	876 (18.1)
61-70	584 (12)
71-80	212 (4.4)
81-90	17 (0.4)
**Total**	**4852 (100)**

Majority of patients presented with right upper abdominal pain (90.36%) of varying duration along with nausea, vomiting, fever, epigastric pain etc.

In the present study, we categorized the nonneoplastic lesions as chronic cholecystitis without any specific findings and chronic cholecystitis with specific findings. Out of 4852 non-neoplastic lesions, cholecystitis without any specific findings were noticed in 804 (16.57%) males and 2224 (45.84%) females. Cholecystitis with specific findings are tabulated and all these findings were more common in females than males (Table 2).

**Table 2:** Distribution of gallbladder lesions with respect to gender (n = 4852).

Diagnosis		Gender	
		Male n (%)	Female n (%)
Cholecystitis without any specific finding		804 (16.57)	2224 (45.84)
	Cholelithiasis	353 (7.28)	1125 (23.19)
	Cholesterolosis	28 (0.58)	97 (2)
	Xanthogranulomatous	6 (0.12)	11(0.23)
Cholecystitis with specific finding	Pyloric metaplasia	48 (0.99)	114 (2.35)
	Intestinal metaplasia	13 (0.27)	29 (0.60)
**Total**		**1252 (25.8)**	**3600 (74.2)**

Chronic cholecystitis without any specific finding was seen in 3028 (62.4%) cases. Chronic cholecystitis with specific findings was seen in 1824 (37.59%) cases which included cholelithiasis (1478 cases, 30.5%), cholesterolosis (125 cases, 2.6%), xanthogranulomaotus lesion (17 cases, 0.4%), pyloric metaplasia (162 cases, 3.3%) and intestinal metaplasia (42 cases, 0.9%) (Table 3).

**Table 3:** Frequency of non-neoplastic lesions of gallbladder.

Diagnosis			n (%)
	Cholecystitis without any specific finding		3028 (62.4)
		Cholelithiasis	1478 (30.5)
Non-neoplastic lesions	Cholecystitis with specific finding	Cholesterolosis	125 (2.6)
		Xanthogranulomatous	17 (0.4)
		Pyloric metaplasia	162 (3.3)
		Intestinal metaplasia	42 (0.9)
	**Total**		**4852 (100)**

Chronic cholecystitis without any specific findings was the most common lesion in age group 41-50 years with 758 (15.6%) cases followed by age group 31-40 years with 608 (12.5%) cases and age group 51-60 years with 539 (11.1%) cases (Table 4).

**Table 4:** Distribution of gallbladder lesions in different age groups (n = 4852).

		Age-group (years) n (%)								
**Diagnosis**		0-10	11-20	21-30	31-40	41-50	51-60	61-70	71-80	81-90
Cholecystitis without any specific finding		10 (0.2)	92 (1.8)	514 (10.5)	608 (12.5)	758 (15.6)	539 (11.1)	363 (7.48)	131 (2.6)	13 (0.2)
	Cholelithiasis	1 (0.02)	51 (1.05)	251 (5.1)	297 (6.1)	381 (7.8)	263 (5.4)	176 (3.6)	56 (1.2)	2 (0.04)
	Cholesterolosis	2 (0.04)	3 (0.06)	26 (0.5)	38 (0.7)	25 (0.5)	22 (0.4)	5 (0.1)	4 (0.08)	0
Cholecystitis with specific finding	Xantho granulo matous	0	2 (0.04)	3 (0.06)	3 (0.06)	1 (0.02)	3 (0.06)	3 (0.06)	0	2 (0.04)
	Pyloric metap lasia	0	3 (0.06)	21 (0.4)	26 (0.5)	28 (0.5)	37 (0.7)	30 (0.6)	17 (0.3)	0
	Intestinal metaplasia	0	2 (0.04)	3 (0.06)	7 (0.1)	7 (0.1)	12 (0.2)	7 (0.1)	4 (0.08)	0
**Total**		**13 (0.26)**	**153 (3.15)**	**818 (16.8)**	**979 (20.1)**	**1200 (24.7)**	**876 (18.05)**	**584 (12.03)**	**212 (4.3)**	**17 (0.35)**

## DISCUSSION

Gallbladder disease is a frequently encountered pathology of the biliary tract. Gallbladder is having a wide spectrum of diseases ranging from congenital anomalies, calculi and its complications, noninflammatory & inflammatory to the neoplastic lesions. So, it is important to classify various histomorphological types of gallbladder lesions.^2^

Damor NT et al and Khanna R et al have reported that majority of non-neoplastic lesions of gallbladder in 3rd to 5th decades of life.^3,5^ Several studies have observed non-neoplastic lesions in >95% of their cholecystectomy specimens.^1,4,6,7^ Present study also showed the non-neoplastic lesions in 4852 (98.73%) cases and maximum number of lesions were noted in 4^th^ to 6^th^ decades of life.

The propensity for cholelithiasis is influenced by a number of factors, including gender, age, ethnicity, obesity, rapid weight loss, smoking, sedentary lifestyle, insulin resistance, and the metabolic syndrome. Cholelithiasis is more common among women than men.^8^ The female hormone estrogen is known to increase the saturation of cholesterol in bile, thus increasing the risk of gallstone formation.^9^ Cholelithiasis can be considered as a major cause of mortality and morbidity throughout the world and produces diverse histopathological changes in gallbladder mucosa which includes acute inflammation, chronic inflammation, granulomatous inflammation, hyperplasia, cholesterolosis, dysplasia and carcinoma.^4^,^10^

Majority of the cases of gallstones are silent.^4^ Gallstones, chronic inflammation, carcinogen exposure and Helicobacter pylori infection are considered as an important etiological factor in carcinogenesis, many of which account for its geographical, ethnic and sex distribution.^9,11,12^ The estimated prevalence of gallstones in gallbladder lesions reported in the neighbouring country India is between 2% to 29%.^13^ Awasthi N, Mushtaq M, et al, Srinivasan G and Sekar SI in their studies found that the gallstones were present in 697 (95.2%), 225(62.5%) and 31(86.11%) cases respectively.^1,2,10^ Present study showed gallstones in 1478 (30.5%) cases of non-neoplastic lesions.

Chronic cholecystitis was the most common findings of gallbladder lesions in studies conducted by Awasthi N, Talreja V, et al, Kafle SU, et al. with 732 (97.1%) cases, 756 (77.69) cases and 25 (50%) cases respectively.^1,14,15^ Cholesterolosis is also one of the findings in cholecystectomy specimens. Chronic cholesystitis with cholesterolosis was observed in studies done by Awasthi N, Mohan H et al and Kafle SU et al in 117 (15.98%), 112 (10.2%), and 11(22%) cases respectively.^1,15,16^ Xanthogranulomatous cholecystitis was observed to be 1.04% to 3% in various studies published previously.^1^,^2^,^10^,^14^,^16^ In the present study, chronic cholecystitis without any specific finding was observed in 3028 (62.4%) cases, chronic cholecystitis with cholesterolosis in 125 (2.6%) cases and xanthogranulomatous cholecystitis in 17 (0.4%) cases.

Gallbladder mucosa shows varying degrees of changes in the lining epithelium like atrophy, hyperplasia and metaplasia in microscopic examination. The metaplasia may be of pyloric type or intestinal type.^5^ Awasthi N and Damor NT, et al. noticed pyloric metaplasia in 0.3% and 1% cases respectively.^1,3^ Few other studies showed both types of metaplasia. Kafle SU, et al. noticed pyloric metaplasia in 16 (33%) cases and intestinal metaplasia in 4 (8%) cases while Khanna R, et al. observed pyloric metaplasia in 4 (20%) cases and intestinal metaplasia in 2 (10%) cases.^5,15^ Similary, pyloric metaplasia was present in 162 (3.3%) cases and intestinal metaplasia in 42(0.9%) cases in the current study. Gallbladder metaplasia has been documented as the precursor lesion of dysplasia and therefore carcinoma.^17^

Unfortunately, our common practice is to discard gallbladder specimen after cholecystectomy. So, histopathological examination of gallbladder specimen is important and is recommended to examine in all the cholecystectomy specimens.^18^

## CONCLUSIONS

The prevalence of non-neoplastic lesions of gallbladder is similar to other studies done in similar setings. Cholecystitis has a wide histomorphological spectrum. Female predominance was observed in non-neoplastic lesions of gallbladder and majority of patients were in fifth decade of life. Incidental findings like xanthogranulomatous changes, pyloric and intestinal metaplasia were also observed in quite a good number of cases.
